# Typhoid Fever in Young Children in Bangladesh: Clinical Findings, Antibiotic Susceptibility Pattern and Immune Responses

**DOI:** 10.1371/journal.pntd.0003619

**Published:** 2015-04-07

**Authors:** Farhana Khanam, Md. Abu Sayeed, Feroza Kaneez Choudhury, Alaullah Sheikh, Dilruba Ahmed, Doli Goswami, Md. Lokman Hossain, Abdullah Brooks, Stephen B. Calderwood, Richelle C. Charles, Alejandro Cravioto, Edward T. Ryan, Firdausi Qadri

**Affiliations:** 1 Centre for Vaccine Sciences, International Centre for Diarrhoeal Disease Research, Bangladesh (icddr,b), Dhaka, Bangladesh; 2 Molecular Microbiology and Microbial pathogenesis program, Division of Biology and Biomedical Sciences, Washington University in St. Louis, Missouri, United States of America; 3 Division of Infectious Diseases, Massachusetts General Hospital, Boston, Massachusetts, United States of America; 4 Department of Medicine, Harvard Medical School, Boston, Massachusetts, United States of America; 5 Avenida Universidad 1900, México, D.F., México; 6 Department of Immunology and Infectious Diseases, Harvard School of Public Health, Boston, Massachusetts, United States of America; University of California San Diego School of Medicine, UNITED STATES

## Abstract

**Background:**

Children bear a large burden of typhoid fever caused by *Salmonella enterica* serotype Typhi (*S*. Typhi) in endemic areas. However, immune responses and clinical findings in children are not well defined. Here, we describe clinical and immunological characteristics of young children with S. Typhi bacteremia, and antimicrobial susceptibility patterns of isolated strains.

**Methods:**

As a marker of recent infection, we have previously characterized antibody-in-lymphocyte secretion (TPTest) during acute typhoid fever in adults. We similarly assessed membrane preparation (MP) IgA responses in young children at clinical presentation, and then 7-10 days and 21-28 days later. We also assessed plasma IgA, IgG and IgM responses and T cell proliferation responses to MP at these time points. We compared responses in young children (1-5 years) with those seen in older children (6-17 years), adults (18-59 years), and age-matched healthy controls.

**Principal Findings:**

We found that, compared to age-matched controls patients in all age cohorts had significantly more MP-IgA responses in lymphocyte secretion at clinical presentation, and the values fell in all groups by late convalescence. Similarly, plasma IgA responses in patients were elevated at presentation compared to controls, with acute and convalescent IgA and IgG responses being highest in adults. T cell proliferative responses increased in all age cohorts by late convalescence. Clinical characteristics were similar in all age cohorts, although younger children were more likely to present with loss of appetite, less likely to complain of headache compared to older cohorts, and adults were more likely to have ingested antibiotics. Multi-drug resistant strains were present in approximately 15% of each age cohort, and 97% strains had resistance to nalidixic acid.

**Conclusions:**

This study demonstrates that *S*. Typhi bacteremia is associated with comparable clinical courses, immunologic responses in various age cohorts, including in young children, and that TPTest can be used as marker of recent typhoid fever, even in young children.

## Author Summary

The highest disease burden of typhoid fever caused by Salmonella enterica serotype Typhi (S. Typhi) is seen in children under five years of age in endemic areas. We investigated both mucosal and systemic immune responses in S. Typhi bacteremic young children (aged, 1 to 5 years) by measuring S. Typhi membrane preparation (MP) specific IgA response in lymphocyte culture secretion and plasma IgA, IgG and IgM responses using ELISA. We also measured T cell proliferation responses using 3H-thymidine incorporation assay. We compared the responses to S. Typhi bacteremic older children (6 to 17 years) and adults (18 to 59 years) and with age-matched healthy controls (HC). Younger children, older children and adults show comparable responses in lymphocyte secretions after onset of illness. Plasma antibody responses to MP vary between young children and other age groups. T cell proliferative responses increased in all age cohorts by late convalescence. Clinical characteristics were similar in all age cohorts. Emergence of MDR S. Typhi strains is seen in young children which does not impact on the clinical symptoms or the immune responses. The results of this study show that natural infections do induce immune response in young children as well as in adults.

## Introduction

Human restricted *Salmonella enterica* serotype Typhi (*S*. Typhi) causes typhoid fever and globally, more than 21 million cases of *S*. Typhi infection and 216,510 deaths due to typhoid fever are reported each year [1]. In endemic areas such as Bangladesh and India, young children under 5 years of age bear a large burden of S. Typhi infection [2–6], although it has been suggested that typhoid fever may be less severe in such young children [7,8]. In reality, the immunological responses and clinical characteristics in young children with *S*. Typhi bacteremia have to date remained poorly characterized. Similarly, it has also been suggested that infection with multi-drug resistant (MDR: resistant to ampicillin, chloramphenicol, trimethoprim/sulfamethoxazole) *S*. Typhi may impact clinical severity and outcome [9], although little is known about MDR *S*. Typhi infection in young children.

To address these issues, we assessed the clinical characteristics of *S*. Typhi bacteremic young children in Dhaka, Bangladesh and determined the antibiotic susceptibility profiles of isolated strains. We compared clinical characteristics between children infected with MDR *S*. Typhi and with non MDR *S*. Typhi. We also assessed humoral and cellular immune responses in *S*. Typhi bacteremic young children, and compared these responses to those in infected older children and adults, as well as to age-matched healthy controls. We assessed humoral responses in plasma, as well as antibodies recovered from circulating lymphocytes secretion using a previously described assay as a marker of recent infection [10,11]. We also characterized the cell mediated immune responses to *S*. Typhi MP antigen in the bacteremic children.

## Materials and Methods

### Enrollment of participants and blood collection

In this study, we enrolled suspected typhoid fever patients from field sites and the Dhaka hospital of the icddr,b based on the criteria of fever of at least 38°C with a minimum duration of 3 days. Blood was collected from patients at the day of enrolment (T1), then at early convalescence 7–10 days later (T2) and at late convalescence 21–28 days (T3) after the *S*. Typhi bacteremia. We categorized our patients into three groups based on their age: Group I (young children; 1–5 years of age; N = 33), Group II (older children; 6–17 years of age; N = 23), and Group III (adults; 18–59 years of age; N = 16). We also collected a single blood sample from healthy controls (N = 20 in each age group) who also resided in the same typhoid endemic area.

### Ethics statement

We obtained informed written consent from all study participants or their guardians prior to study enrollment. This study was approved by the Research Review Committee (RRC) and the Ethical Review Committee (ERC) of the icddr,b and Institutional Review Board of the Massachusetts General Hospital.

### Blood culture and susceptibilities

Blood was cultured using a BacT/Alert automated system, and positive cultures were characterized using standard bacteriological procedures as previously described [11]. Antimicrobial susceptibility profiles were assessed by the disc diffusion method and resistance was determined per Clinical and Laboratory Standards Institute (CLSI) guideline as needed [12]. The susceptibility to azithromycin was determined following the zone size used for Enterobacteriaceae [13].

### Preparation of antibodies recovered from circulating lymphocytes

We recovered antibodies secreted by peripheral lymphocytes (the TPTest; antibody in lymphocyte secretion) as previously described [10,14]. Briefly, we diluted whole blood with equal volume of phosphate-buffered saline (PBS), and added the sample on Ficoll-Isopaque (Pharmacia, Upsala, Sweden), centrifuging at 772×g for 25 min. Plasma was separated from the top and stored at -20°C for use for plasma antibody analyses. We then collected and washed the PBMCs, resuspended the cells in RPMI (Gibco, Gaithersburg, MD) complete medium at a concentration of 1×10^7^ cells per ml of medium, and incubated the samples at 37°C in a 5% CO_2_ incubator for 48 hours. We then collected the secretion and stored the samples at -70°C until antibody analyses was carried out by enzyme linked immunosorbent assay (ELISA) [10].

### Antigen preparation and measurement of antibodies in plasma and using the TPTest

*S*. Typhi specific membrane preparation (MP) antigen was used for the measurement of antibodies (IgA antibody-in-lymphocyte secretion, and IgA, IgG and IgM antibodies in plasma) by ELISA. The MP antigen was prepared from strain *S*. Typhi Ty21a as described previously [15]. In brief, we cultured strain Ty21a on horse blood agar plates and harvested with Tris buffer (10 mM Tris [pH 8.0], 5 mM MgCl2). We then sonicated the mixture, followed by differential centrifugation, and determined the protein content using the Bio-Rad protein assay [16]. We coated microtiter plates (Nunc F, Denmark) with MP antigen (2 μg/ml for plasma, 10 μg/ml for lymphocyte secretion) [14] at room temperature overnight. Following blocking with 1% bovine serum albumin in PBS, we added 100 μl of lymphocyte secretion (1:2 in 0.1% BSA in PBS-Tween for IgA) or plasma (1:25 dilution for IgA and IgM, and 1:50 dilution for IgG), and incubated the plates for 90 min at 37°C. We then washed the plates with 0.05% PBS-Tween, and added horseradish peroxidase-conjugated antibodies to either human IgA, IgG (Jackson Laboratories, Bar Harbor, ME) or IgM (Southern Biotech, Birmingham, AL) (1:1000 in 0.1% BSA in PBS-Tween for IgA, IgG and IgM). We developed the plates with ortho-phenylene diamine (Sigma Chemical Co., St. Louis, MO) in 0.1M sodium citrate buffer and 0.1% hydrogen peroxide. We read the plates kinetically at 450 nm for five minutes at 19-second intervals, and expressed the maximal rate of optical density (OD) change as milli-optical density absorbance units per minute (mAB/min) [14]. We used pooled convalescent plasma from previous patients with known typhoid fever as a positive control on each plate to correct for variations between plates and different days of testing, and divided kinetic reading by this pool, expressing results as ELISA units as previously described [10,14].

### T-cell proliferation assay

For T cell proliferation assays, we re-suspended isolated PBMCs in DMEM complete medium [DMEM/F12 medium (Gibco, GlutaMAX) supplemented with 1% gentamicin and 5% human AB serum] at a concentration of 1x10^6^ cells/ml of medium. We used MP antigen (5 μg/ml) for the stimulation of the cells in tissue culture plates. We used phytohaemagglutinin (PHA) [Murex Diagnostics Ltd, Temple Hill, UK] as a positive control, and keyhole limpet hemocyanin (KLH) as a negative control. We incubated plates at 37°C with 5% CO_2_ for 5 days. From the top of each well, 100 μl of the medium was replaced with fresh medium without disturbing the cells at the bottom after 48 hours of incubation. We added ^3^H-thymidine (1 μCi) to each well on the 5^th^ day of incubation, and incubated the plates for an additional eight hours after which cells were harvested (Skatron instruments, Norway) in Bray’s scintillation fluid (Ultimagold, PerkinElmer, Boston, MA), and ^3^H-thymidine incorporation was assessed using a liquid scintillation β-counter (Beckman LS6500 multipurpose scintillation counter, USA) [17–19]. We expressed results as counts per minute (cpm), and calculated stimulation indices to determine the responses using specimens collected at T2 and T3 [17,20].

### Statistical analyses

Analysis of data and preparation of figures used statistical software SigmaStat (version3.1), Prism4 and Epi Info7 (Centers for Disease Control and Prevention). Statistical evaluation of differences between study days was performed using the Wilcoxon Signed Rank test, and among groups was performed using the unpaired *t* test. Results were considered statistically significant if *p*<0.05.

## Results

### Clinical characteristics of patients with typhoid fever

Among 33 *S*. Typhi bacteremic young children, 8 (24%) patients reported prior antibiotic use, and the median temperature and mean duration of fever were 39.1°C and 4.6 days, respectively (Table 1). Out of the total 72 *S*. Typhi bacteremic patients, significantly higher number of adults had a history of antibiotic taken before enrolment than older children (*n* = 6, 38% vs. *n* = 2, 9%; *p* = 0.028). No significant differences were found in median temperature or duration of fever between young children and older age groups. Although there was a significant difference in the median pulse rate among groups, the values reflected age variation in pulse rate irrespective of illness.

**Table 1:** Baseline information and clinical characteristics of patients with typhoid fever.

Parameters	Young children	Older children	Adults
**Baseline Information**			
Number of *S*. Typhi bacteremic patients	33	23	16
No. of males (%)	19 (58)	10 (43)	4 (25)
Median age in year (25th, 75th centile)	3.2 (2.4, 4.1)	8.4 (6.3, 11.6)	28.4 (24.4, 34.5)
Median temperature in °C (25th, 75th centile)	39.1 (39.0, 39.4)	39.0 (38.5, 39.3)	39.0 (38.5, 39.2)
Duration of fever, days (mean ± standard deviation)	4.6 ± 1.2	4.1 ± 1.2	5.0 ± 0.8
Median pulse/min (25th, 75th centile)	130 (120, 140)	110 (100, 119.5)^a^	90 (88, 92)^a^ ^b^
No. of patients receiving antibiotic before enrolment (%)	8 (24)	2 (9)	6 (38)^b^
**Clinical features**			
No. of patients (%) with:			
Diarrhea	10 (30)	9 (39)	6 (38)
Abdominal pain	7 (21)	5 (22)	3 (19)
Loss of appetite	19 (58)	7 (30)^a^	6 (38)
Headache	4 (12)	8 (35)^a^	10 (63)^a^
Myalgia	6 (15)	4 (17)	13 (81)^a^ ^b^
Rash	0 (0)	0 (0)	0 (0)
Coated tongue	6 (18)	6 (26)	6 (38)
Tender abdomen	2 (6)	0 (0)	0 (0)
Hepatomegaly	0 (0)	0 (0)	0 (0)
Splenomegaly	0 (0)	0 (0)	0 (0)

^a^ *p* < 0.05 when compared to young children

^b^ *p* < 0.05 when compared to older children

Young patients presented with a number of the classical features of typhoid fever, including high grade fever (median temperature: 39.1°C), abdominal pain (*n* = 7, 21%), loss of appetite (*n* = 19, 58%) and coated tongue (*n* = 6, 18%). The number of patients reporting myalgia was significantly higher in adults than younger children (*n* = 13, 81% vs. *n* = 6, 15%; *p* = 0.00002) and older children (*n* = 13, 81% vs. *n* = 4, 17%; *p* = 0.00007). A significantly lower proportion of young patients communicated complaints of headache compared to older children (*n* = 4, 12% vs. *n* = 8, 35%; *p* = 0.04) and adults (*n* = 4, 12% vs. *n* = 10, 63%; *p* = 0.0002); whereas the number of patients with loss of appetite was higher in young children compared to older children (*n* = 19, 58% vs. *n* = 7, 30%; *p* = 0.04). All study participants were treated with antimicrobials and hospitalized at the discretion of the attending physician. All patients recovered, and none developed severe complications such as intestinal hemorrhage or perforation, or encephalopathy as they were appropriately treated.

### Antibiotic susceptibility profiles of strains isolated from patients with *S*. Typhi bacteremia

In young children, the number of isolates with resistance to ampicillin, chloramphenicol and co-trimoxazole was 13 (39%), 10 (30%) and 10 (30%), respectively; and 5 (15%) isolated strains were MDR. Of 23 *S*. Typhi strains isolated from older children, 6 (26%), 3 (13%) and 3 (13%) were resistant to ampicillin, chloramphenicol and co-trimoxazole, respectively; and 3 (13%) were MDR. Among 16 isolates from adults, 2 (13%) were MDR strains. All 72 (100%) strains isolated from the three age groups were sensitive to cephalosporins (ceftriaxone and cefixime) and to azithromycin, although 70 (97%) strains had reduced susceptibility to ciprofloxacin and resistance to nalidixic acid, with all the isolates in young children having reduced susceptibility to ciprofloxacin (Table 2). No isolate was sensitive for ciprofloxacin. We found no significant differences in the number of patients requiring hospitalization nor in other clinical characteristics between MDR and non MDR *S*. Typhi infections in either young children or the older age groups. We also compared the clinical characteristics between nalidixic acid resistant (NAR) and non-NAR isolates and did not find any difference.

**Table 2:** Antibiotic susceptibility pattern of isolated strains from the patients of three age groups.

Antibiotics	Young children (n = 33)	Older children (n = 23)	Adults (n = 16)
Resistance to ampicillin	13 (39)	6 (26)	2 (13)
Resistance to chloramphenicol	10 (30)	3 (13)	2 (13)
Resistance to co-trimoxazole	10 (30)	3 (13)	2 (13)
MDR*	5 (15)	3 (13)	2 (13)
Resistance to nalidixic Acid	33 (100)	23 (100)	14 (88)^a^
Reduced susceptibility to ciprofloxacin	33 (100)	23 (100)	14 (88)^a^
Resistance to ceftriaxone	0 (0)	0 (0)	0 (0)
Resistance to cefixime	0 (0)	0 (0)	0 (0)
Resistance to azithromycin	0 (0)	0 (0)	0 (0)

Results are n (%).

*MDR, multidrug-resistant (resistant to ampicillin, chloramphenicol and co-trimoxazole).

^a^ *p* < 0.05 when compared to young children.

### *S*. Typhi specific MP-IgA responses in lymphocyte secretions

**Fig 1:**
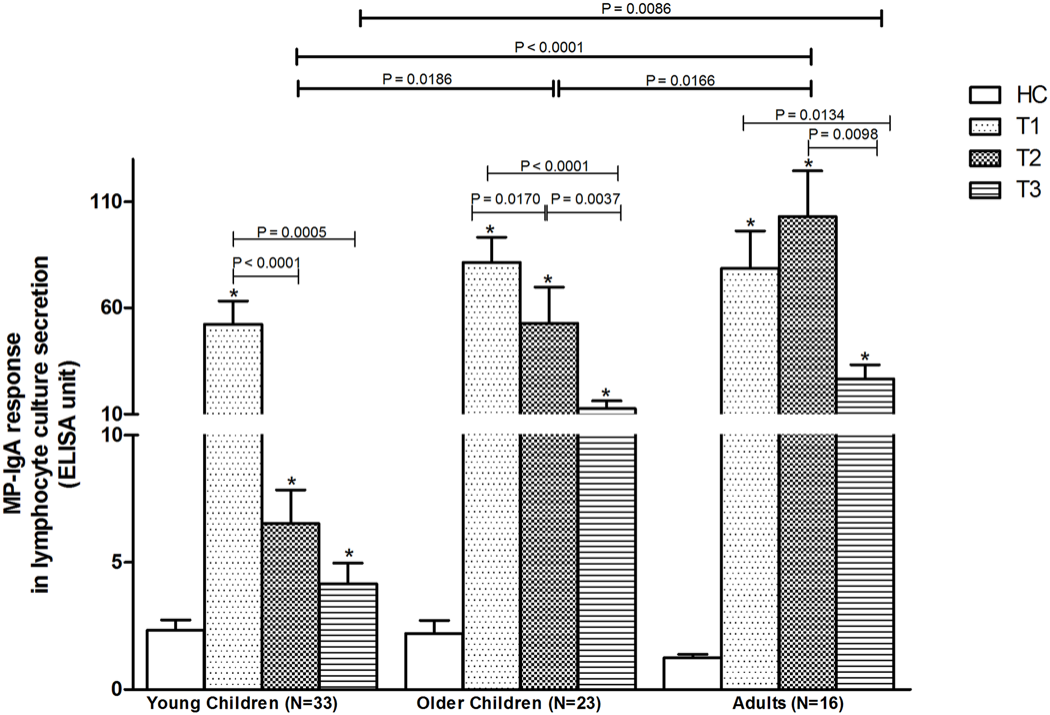
MP-IgA responses in lymphocyte culture secretion in patients with *S*. Typhi bacteremia. Mean with standard error of mean (SEM) are shown for T1 (at day of enrolment), T2 (at early convalescence: 7–10 days after enrolment) and T3 (at late convalescence: 21–28 days after enrolment). Statistical difference between patients and age-matched healthy control (HC): *. MP: *S*. Typhi membrane preparation.

At enrollment (T1), young children had significantly higher MP-IgA responses in lymphocyte secretions than at early (T2) and late (T3) convalescent stages (Fig. 1). The responses in young healthy control children were significantly lower than in the patients at all three stages of the infection (*p*<0.0001). A sub-analysis of responses when stratified by ingestion of antibiotics prior to presentation showed similar results. *S*. Typhi bacteremic young children had similar MP-IgA responses in lymphocyte secretions as did older children and adults at the early stage of the disease (T1). The differences in response were seen mainly at later stages of the disease (T2 and T3). At T2, there was a significantly higher response in older compared to younger children (*p* = 0.0186). The responses in young children were also lower than those in adults at T2 (*p*< 0.0001) and at T3 (*p* = 0.0086). The patients in all age groups had significantly higher responses (*p*< 0.0001) in lymphocyte secretions than in those of their age-matched healthy controls (Fig. 1).

### Plasma antibody responses to *S*. Typhi specific MP in children and adult patients with typhoid fever

**Fig 2:**
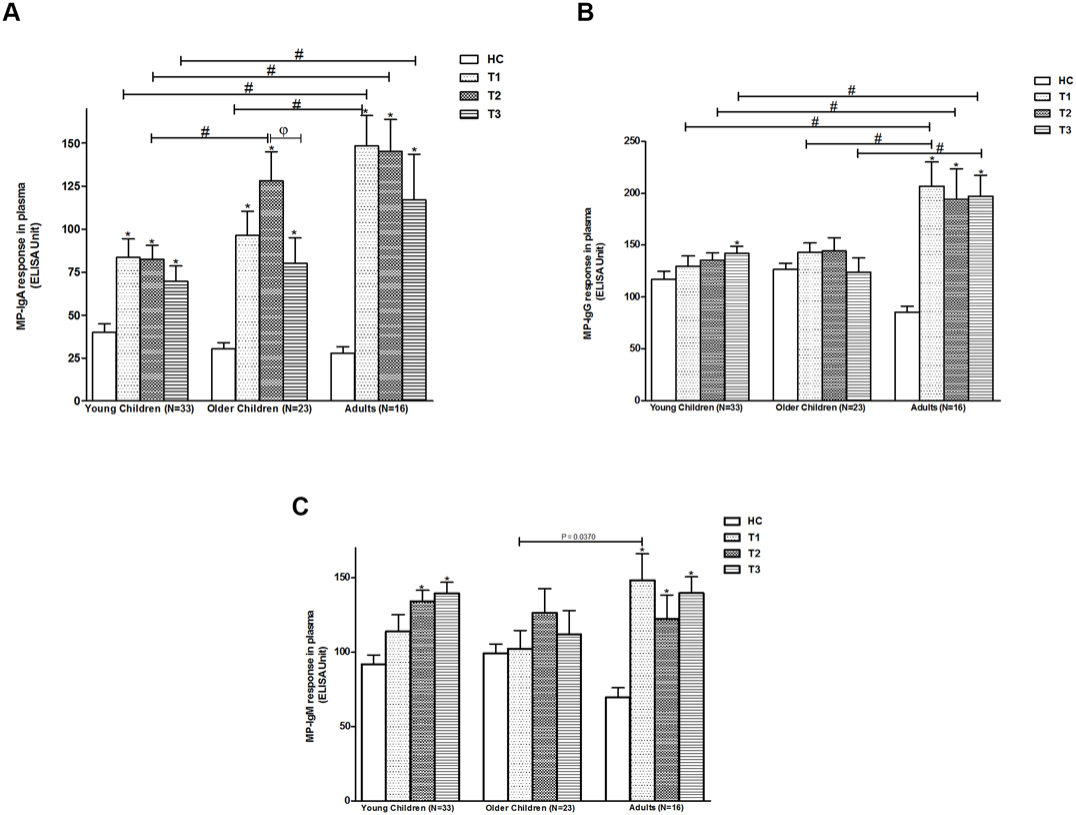
Plasma antibody responses to MP in patients with *S*. Typhi bacteremia. Anti-MP-IgA (2a), anti-MP-IgG (2b) and anti-MP-IgM (2c) responses are shown as mean with standard error of mean (SEM) for T1 (at day of enrolment), T2 (at early convalescence: 7–10 days after enrolment) and T3 (at late convalescence: 21–28 days after enrolment). Statistical difference between patients and age-matched healthy control (HC): *. MP: *S*. Typhi membrane preparation.

In plasma, young children had significantly higher MP-IgA responses at all time points compared to age-matched healthy controls, similar to both older cohorts. The plasma values were highest in adults (Fig. 2a). In the plasma-MP-IgG analyses, adults showed higher responses than healthy controls at all three time points, while there were no statistically different increases in MP IgG responses compared to age-matched healthy controls in either younger or older children, except for a slight increase in MP IgG at T3 in the younger children (Fig. 2b).

The plasma-MP-IgM analyses were similar to the plasma IgG results. Adults showed significantly higher responses at all three time points compared to age-matched healthy controls, while there were no significant increases in older children at any time point, and small increases at T2 and T3 in younger children (Fig. 2c).

### T cell proliferative response in patients with *S*. Typhi bacteremia

**Fig 3:**
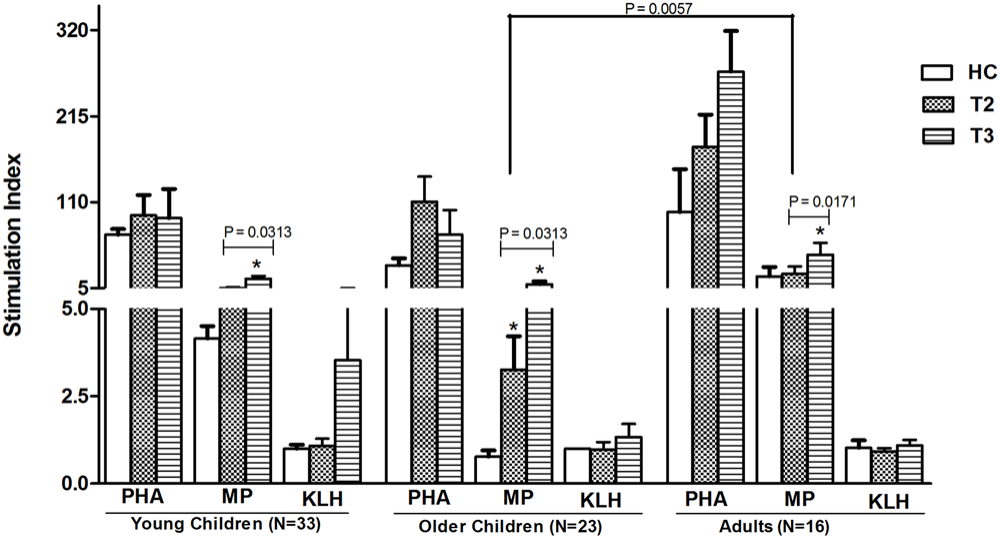
T-cell proliferation responses in patients with *S*. Typhi bacteremia. T-cell proliferation responses against *S*. Typhi specific membrane preparation (MP), control proteins phytohaemagglutinin (PHA) and keyhole limpet hemocyanin (KLH) at early and late convalescent stages of disease in *S*. Typhi bacteremic patients and in healthy controls (HC). Stimulation index was calculated as the ratio of net cpm with antigen to net cpm without antigen (only media). Mean with standard error of mean (SEM) are shown for T1 (at day of enrolment), T2 (at early convalescence: 7–10 days after enrolment) and T3 (at late convalescence: 21–28 days after enrolment). Statistical difference between patients and age-matched healthy control (HC): *.

We compared T cell proliferative responses to *S*. Typhi MP antigen in young healthy controls and in children with typhoid fever at early (T2) and late (T3) convalescence (Fig. 3). T cell proliferative assays following *S*. Typhi MP stimulation increased in all age cohorts by late convalescence. We did not detect any difference in proliferative indices between healthy controls and children at early convalescence, but infected children had significantly higher proliferative responses at late convalescence compared to healthy controls (*p* = 0.001) and compared to early stage of convalescence (*p* = 0.038). We found no difference in T cell proliferative responses to *S*. Typhi MP antigen in early (T2) and late convalescent stage (T3) of the disease between bacteremic young children and older age groups (Fig. 3).

## Discussion

This study characterizes humoral and cellular immune responses during typhoid fever with confirmed *S*. Typhi bacteremia, the clinical picture of the disease, and the current antibiotic susceptibility patterns of *S*. Typhi strains in young children in Dhaka, Bangladesh. This age group would be a primary target for typhoid control and vaccination programs. Our main finding was that young children had comparable clinical, microbiologic and immune features compared to those seen in older children and adults. These observations support the use of typhoid control and vaccination strategies in children under five years of age. In particular, our observation that approximately 15% of *S*. Typhi isolates were multi drug resistant and that resistance to nalidixic acid and decreased susceptibility to ciprofloxacin are now common in Dhaka, including in the youngest children, suggest that options for antimicrobial therapy are becoming limited, and should be viewed as a strong reason to support typhoid control and vaccination programs. Although no complications due to typhoid fever were seen in our study patients, the clinical features of *S*. Typhi infection in young children was similar to those seen in older children and adults, the age cohorts traditionally thought to bear the largest burden of disease [7,8,21].

Similarly, we were unable to find an association between infection with MDR strains and disease severity, although previous studies [22–24] report such an association. Our finding, however, is in agreement with a study carried out in New Delhi [2].

Our study includes one of the first analyses of the range of immune responses following typhoid fever in young children. We have previously shown that antibodies secreted by lymphocytes circulating in the peripheral blood of adults with typhoid and paratyphoid fever increase transiently during typhoid fever, and that these responses can be used as both a sensitive and specific diagnostic assay to identify patients with enteric fever [10,14]. These activated lymphocytes may represent cells activated early in infection either at the mucosal surface or in systemic sites. Our results suggest that the lymphocytes of young children with *S*. Typhi bacteremia are similarly activated as those of older children and adults. Our results also suggest that an assay such as the previously described Typhoid Paratyphoid Test (TPTest), which is based on assessing antibodies secreted by circulating lymphocytes, can be used to identify young children with typhoid fever. The results in this current report suggest that this assay can distinguish infected from non-infected children in this endemic zone, and that the transient nature of the response (falling to baseline by late convalescence) would support its further development as a diagnostic assay early in disease, to assist with disease burden analyses, and to help target and assess control and vaccination programs. We found higher response in older children and adults to MP stimulation in lymphocyte culture supernatant at later stages of the disease (T2 and T3 time points) which may be due to previous exposure and specific immunity developed to *S*. Typhi. Our results also suggest that the plasma IgA response against *S*. Typhi membrane preparation could also be used to identify acutely infected patients. However, the magnitude of these responses was appreciably lower in young children compared to adults which may be also due to previous exposure. Our results suggest that IgG and IgM responses against MP would have limited clinical utility in endemic setting.

We have previously characterized the cellular immune responses in adult patients with typhoid fever [17]. Our current analysis suggests that even young children develop proliferative responses to typhoid antigens by late convalescence. Significant responses to *S*. Typhi MP even in healthy adults also suggest that baseline proliferative responses may increase over time in this typhoid endemic area. The reason for lower stimulation index among healthy control in older children compared to young children and adults is not currently clear.

Our study has a number of limitations. We (1) only assessed immune responses against a crude *S*. Typhi antigen preparation as in our earlier studies [10,11] and not purified antigens; (2) we used a proliferative assay to characterize cellular immunity and did not further characterize specific immune mechanistic pathways; (3) our study was only of moderate size; and (4) our study was hospital-based and did not involve active community-based case detection. Despite these limitations, our study is the largest to date characterizing humoral and cellular immune responses, clinical features and microbiologic susceptibility profiles in young children with bacteremic typhoid fever. Our results suggest that young children with *S*. Typhi bacteremia develop a clinical illness and host immune responses comparable to those seen in older children and adults. Our results also support the further development of the TPTest as a diagnostic assay for enteric fever, for use in estimating disease burden and the effects of control and vaccination programs across the different age cohorts.

## Supporting Information

S1 Checklist STROBE checklist. (DOC)
