# Supplementary material for: Typhoid Fever in Young Children in Bangladesh: Clinical Findings, Antibiotic Susceptibility Pattern and Immune Responses
Source: PLoS Negl Trop Dis. 2015 Apr 7;9(4):e0003619. doi: 10.1371/journal.pntd.0003619 (PMC4388457; doi:10.1371/journal.pntd.0003619)
Supplement: S1 Checklist — (DOC) [file pntd.0003619.s001.doc]

**STROBE Checklist for PNTD-D-14-02019R1: Typhoid fever in young children in Bangladesh: clinical findings, antibiotic susceptibility pattern and immune responses**

|  | Item No | Recommendation |
| --- | --- | --- |
| **Title and abstract** | 1 | (*a*) Indicate the study’s design with a commonly used term in the title or the abstract  **Response:** Title- Typhoid fever in young children in Bangladesh: clinical findings, antibiotic susceptibility pattern and immune responses (Page:01) |
|  |  | (*b*) Provide in the abstract an informative and balanced summary of what was done and what was found  **Response:** Provided in the abstract section (Page: 02) |
| Introduction | | |
| Background/rationale | 2 | Explain the scientific background and rationale for the investigation being reported  **Response:** Page 04, Line 58-67 |
| Objectives | 3 | State specific objectives, including any prespecified hypotheses  **Response:** Page 04, Line 69-77 (No prespecified hypothesis) |
| Methods | | |
| Study design | 4 | Present key elements of study design early in the paper  **Response:** Described in the Abstract section (Page no. 02) |
| Setting | 5 | Describe the setting, locations, and relevant dates, including periods of recruitment, exposure, follow-up, and data collection  **Response:** Page 05, Line 82-89 |
| Participants | 6 | (*a*) Give the eligibility criteria, and the sources and methods of selection of participants  **Response:** Page 05, Line 83 |
| Variables | 7 | Clearly define all outcomes, exposures, predictors, potential confounders, and effect modifiers. Give diagnostic criteria, if applicable  **Response:** Exposure to *S*. Typhi infection and clinical outcome of the patients (Page 9, Line 179-191). Diagnostic criteria: The patients were selected based on the result of blood culture. |
| Data sources/ measurement | 8* | For each variable of interest, give sources of data and details of methods of assessment (measurement). Describe comparability of assessment methods if there is more than one group  **Response:** We have incorporated the results of ELISA method using the lymphocyte culture secretion and plasma and also inserted the data of T cell proliferation assay |
| Bias | 9 | Describe any efforts to address potential sources of bias  **Response:** We have carried out the experiments in patients based on the result of blood culture and there was no bias in the study |
| Study size | 10 | Explain how the study size was arrived at  **Response:** In this study we have measured the immune responses in the specimens of blood culture confirmed *S*. Typhi bacteremic patients of different age groups |
| Quantitative variables | 11 | Explain how quantitative variables were handled in the analyses. If applicable, describe which groupings were chosen and why |
| Statistical methods | 12 | (*a*) Describe all statistical methods, including those used to control for confounding  **Response:** Page 8, Line 150-154 |
|  |  | (*b*) Describe any methods used to examine subgroups and interactions |
|  |  | (*c*) Explain how missing data were addressed |
|  |  | (*d*) If applicable, describe analytical methods taking account of sampling strategy |
|  |  | (*e*) Describe any sensitivity analyses |
| Results | | |
| Participants | 13* | (a) Report numbers of individuals at each stage of study—eg numbers potentially eligible, examined for eligibility, confirmed eligible, included in the study, completing follow-up, and analysed  **Response:** Page 05, line 85-89 |
|  |  | (b) Give reasons for non-participation at each stage |
|  |  | (c) Consider use of a flow diagram |
| Descriptive data | 14* | (a) Give characteristics of study participants (eg demographic, clinical, social) and information on exposures and potential confounders  **Response:** Page 8-9, line 157 - 176 |
|  |  | (b) Indicate number of participants with missing data for each variable of interest  **Response:** No missing data |
| Outcome data | 15* | Report numbers of outcome events or summary measures  **Response:**  *S*. Typhi specific MP-IgA responses in lymphocyte secretions (Page 9 - 10, Line 194 -205, and also Figure 1);  Plasma antibody responses to *S.* Typhi specific MP in children and adult patients with typhoid fever (Page 10-11, Line 208-217, and also Figure 2a, 2b, 2c);  T cell proliferative response in patients with *S*. Typhi bacteremia (Page 11, Line 219-227, and also Figure 3) |
| Main results | 16 | (*a*) Give unadjusted estimates and, if applicable, confounder-adjusted estimates and their precision (eg, 95% confidence interval). Make clear which confounders were adjusted for and why they were included  **Response:** We only used unadjusted estimates in this study |
|  |  | (*b*) Report category boundaries when continuous variables were categorized |
|  |  | (*c*) If relevant, consider translating estimates of relative risk into absolute risk for a meaningful time period  **Response:** Not relevant for our study |
| Other analyses | 17 | Report other analyses done—eg analyses of subgroups and interactions, and sensitivity analyses  **Response:** Not applicable for our study |
| Discussion | | |
| Key results | 18 | Summarise key results with reference to study objectives  **Response:** Page 11-13, line 229 - 273 |
| Limitations | 19 | Discuss limitations of the study, taking into account sources of potential bias or imprecision. Discuss both direction and magnitude of any potential bias  **Response:** Page 13, line 274 - 278 |
| Interpretation | 20 | Give a cautious overall interpretation of results considering objectives, limitations, multiplicity of analyses, results from similar studies, and other relevant evidence  **Response:** Page 13, line 279 - 283 |
| Generalisability | 21 | Discuss the generalisability (external validity) of the study results  **Response:** Page 13 and 14, line 283 - 285 |
| Other information | | |
| Funding | 22 | Give the source of funding and the role of the funders for the present study and, if applicable, for the original study on which the present article is based  **Response:** Source of funding was listed during the submission process. To avoid reiteration we did not mention that in the text. The funders had no role in study design, data collection, analysis and publication. There is no conflict of interest. |

*Give information separately for exposed and unexposed groups.

**Note:** An Explanation and Elaboration article discusses each checklist item and gives methodological background and published examples of transparent reporting. The STROBE checklist is best used in conjunction with this article (freely available on the Web sites of PLoS Medicine at http://www.plosmedicine.org/, Annals of Internal Medicine at http://www.annals.org/, and Epidemiology at http://www.epidem.com/). Information on the STROBE Initiative is available at www.strobe-statement.org.
